# Polyphenol-Rich Leaf of *Annona squamosa* Stimulates Insulin Release from BRIN-BD11 Cells and Isolated Mouse Islets, Reduces (CH_2_O)_n_ Digestion and Absorption, and Improves Glucose Tolerance and GLP-1 (7-36) Levels in High-Fat-Fed Rats

**DOI:** 10.3390/metabo12100995

**Published:** 2022-10-20

**Authors:** Prawej Ansari, J.M.A. Hannan, Veronique Seidel, Yasser H.A. Abdel-Wahab

**Affiliations:** 1 Department of Pharmacy, School of Pharmacy and Public Health, Independent University, Bangladesh (IUB), Dhaka 1229, Bangladesh; 2 School of Biomedical Sciences, Ulster University, Coleraine BT52 1SA, UK; 3 Natural Products Research Laboratory, Strathclyde Institute of Pharmacy and Biomedical Sciences, University of Strathclyde, Glasgow G4 0RE, UK

**Keywords:** diabetes, insulin, glucose, phytoconstituents, *Annona squamosa*, GLP-1

## Abstract

*Annona squamosa*, commonly known as custard apple, is traditionally used for the treatment of various diseases including diabetes, cardiovascular disease (CVD), and gastritis. This study was undertaken to investigate the effects of an ethanolic (80% *v*/*v*) extract of *A. squamosa* (EEAS) leaves in vitro on insulin secretion from clonal pancreatic BRIN BD11 β-cells and mouse islets, including mechanistic studies on the effect of EEAS on membrane potential and intracellular calcium ion concentration. Additional in vitro glucose-lowering actions were assessed. For in vivo studies, high-fat-fed (HFF) obese/normal rats were selected. EEAS increased insulin secretion in vitro in a dose-dependent manner. This effect was linked to β-cell membrane depolarisation and cytoplasmic Ca^2+^ influx. In the presence of isobutyl methylxanthine (IBMX), tolbutamide, or KCl, the insulin-releasing effect of EEAS was increased, suggesting its effect was also mediated via a K_ATP_-independent pathways. EEAS inhibited insulin glycation, glucose absorption, and DPP-IV enzyme activity in vitro and enhanced glucose uptake and insulin action in 3T3L1 cells. In vivo, gut motility, food intake, glucose tolerance, plasma insulin, and active GLP-1 (7-36) levels were improved, whereas plasma DPP-IV levels were reduced in HFF rats. EEAS attenuated the absorption of sucrose and glucose as well as decreased serum glucose levels after sucrose loading and in situ intestinal perfusion in non-diabetic rats. Rutin, proanthocyanidin, and squafosacin G were putatively identified as the anti-hyperglycaemic phytomolecules in EEAS using HPLC followed by LC-MS analysis. This study illustrates the potential of *A. squamosa* and its phytoconstituents as a source of potential antidiabetic agents.

## 1. Introduction

Diabetes mellitus is one of the most prevalent metabolic syndromes caused due to deficits in insulin secretion and/or insulin resistance [1]. The International Diabetes Federation (IDF) has estimated that 500 million people worldwide are diabetic, with this number expected to rise to 783.2 million in the next 23 years [2]. The three major categories of diabetes include type 1, type 2, and gestational diabetes. Type 1 (insulin-dependent diabetes mellitus) is characterised by the close to total destruction of the β-cells of the pancreatic islets [3], while type 2 (non-insulin-dependent diabetes mellitus) is induced due to obesity, insulin resistance and/or impaired insulin secretion [4]. The rapidly increasing prevalence of type 2 diabetes mellitus (T2DM) is primarily attributable to obesity and physical inactivity, making it the most prevalent form worldwide [5]. An imbalance in energy consumption leads to obesity, and this causes an increase in oxidative stress. The latter interferes with glucose metabolism, leading to insulin resistance and eventually to the development of T2DM [6]. Hyperglycaemia caused by diabetes leads to long-term complications such as cardiovascular disease, peripheral and cerebrovascular disease, neuropathy, retinopathy, and nephropathy, which may ultimately result in end-stage organ damage causing cardiac arrest, stroke, blindness, and renal failure [7,8,9].

A healthy diet, weight management, and regular exercise are the first steps to lowering blood sugar levels [10]. Additionally, to increase insulin secretion and improve its function, one or more oral anti-hyperglycaemic medications, such as biguanides, sulphonylureas, thiazolidinediones, SGLT-2 inhibitors, meglitinides, α-glucosidase inhibitors, DPP-IV inhibitors, GLP-1 analogues, GIP, and GLP-1 receptor co-agonists, and/or synthetic insulin, may be used [11,12,13]. However, in addition to being expensive and unavailable to people living in remote and poor areas, these synthetic drugs are associated with a number of adverse effects, including gastrointestinal disorders, weight gain, renal problems, liver diseases, and hypoglycaemia [11,14,15,16]. Thus, extensive research is being conducted on plant- and animal-based resources to develop alternative medicines for the treatment and management of this global epidemic. Due to their multiple health benefits, medicinal plants have been traditionally used to treat diseases in local communities since ancient times [17]. To date, over 800 plant species have been identified as having anti-diabetic properties [18]. Alkaloids, polyphenols, tannins, flavonoids, and other phytochemicals are among the many phytoconstituents found in these plants that are highly potent and effective in lowering blood glucose, and thus may help in the discovery and development of newer drugs to combat T2DM [17,19].

Many of these phytoconstituents inhibit DPP-IV action, which has been the focus of recent research into antidiabetic medicines. The enzyme dipeptidyl peptidase IV (DPP-IV) is responsible for the degradation of incretin hormones, which are generated by the gut after meals [20]. The cyclic adenosine monophosphate (cAMP) pathway is triggered upon the interaction of such hormones with certain pancreatic β-cell receptors. This promotes insulin secretion from β-cells and thus, incretin hormones contribute significantly to the control of postprandial hyperglycaemia [21]. Hence, plants containing compounds with DPP-IV inhibitory activity are widely sought for.

*Annona squamosa*, a member of the Annonaceae family, is a tropical tree growing in many South Asian countries including Bangladesh [14,22,23]. The leaves, fruits, roots, bark, and seeds of *A. squamosa* are employed to treat diabetes, gastritis, diarrhoea, dysentery, malaria, parasitic infections, and several other health complications. Moreover, recent findings have documented the antidiabetic, hepatoprotective, and antioxidative effects of *A. squamosa*, which contains phytomolecules such as quercetin, rutin, kaempferol, and β-caryophyllene [14,24]. In vitro studies with *A. squamosa* hot water extract revealed a reduction in protein glycation, DPP-IV enzyme activity, and glucose absorption. In HFF obese rats, it improved glucose tolerance and plasma insulin levels and β-cell functions [14]. The current study aims to determine the in vitro and in vivo anti-hyperglycaemic activity of the ethanol extract of *A. squamosa* (EEAS) along with its mechanism of action in the management of diabetes.

## 2. Materials and Methods

### 2.1. Collection and Preparation of Plant Extracts

*Annona squamosa* leaves were collected from the University Ayurvedic Research Center (UARC), Jahangirnagar University, Dhaka, Bangladesh. The plant was identified by a botanical taxonomist at the Bangladesh National Herbarium, Mirpur, Dhaka, and was assigned accession number 43,754 [14]. The collected leaves were washed, shade-dried, and then pulverised to a fine powder. The dried powdered leaves (200 g) were macerated in 80% ethanol (1 L) and the mixture was shaken in an orbital shaker for 48–72 h at a rotational speed of 550 rpm. Following filtration using Whatman filter paper, the filtrate was concentrated to dryness using a rotary evaporator (BibbyRE-200, Sterilin Ltd., Newport, UK). The sticky residue was freeze-dried using a freeze dryer (Varian 801 LY-3-TT, Varian, Lexington, MA, USA) and stored at 4 °C for further studies [13,25].

### 2.2. In Vitro Insulin-Releasing Studies

The insulin-releasing effects of EEAS were tested using clonal pancreatic BRIN-BD11 β-cells and isolated mouse islets. The BRIN-BD11 cells were treated with various concentrations of EEAS or insulin secretagogues in the presence or absence of glucose (1.1, 5.6, or 16.7 mM) and were incubated at 37 °C for 20 min in a CO_2_ incubator [14]. Pancreatic islets were extracted from mice using collagenase P isolated from *Clostridium histolyticum* (Sigma-Aldrich, Dorset, UK), and were subsequently cultured for 48 h. The islets were incubated with 16.7 mM glucose at 37 °C for 1 h with/without EEAS, alanine, and GLP-1 in a CO_2_ incubator. The supernatants were separated by centrifugation, and then preserved at −20 °C for insulin radioimmunoassay. The secretory pathways activated by EEAS were identified by assessing the effects of EEAS in the presence of insulin secretagogues or inhibitors such as diazoxide (a K_ATP_ channel opener), tolbutamide (a sulphonylurea and K_ATP_ channel blocker), verapamil (a voltage-dependent Ca^2+^ channel blocker), IBMX (a phosphodiesterase inhibitor), 30 mM KCl, and 10 mM alanine. KCl and alanine cause plasma membrane depolarisation and Ca^2+^ influx. Alanine does this mostly by co-transport with Na+ and metabolism with ATP production [11,14].

### 2.3. Membrane Potential and Intracellular Calcium Concentration ([Ca^2^^+^]_i_)

A fluorometric imaging plate reader (FLIPR) membrane potential and [Ca^2+^]_i_ assay kit (Molecular Devices, Sunnyvale, CA, USA) were employed to examine the effects of EEAS on the membrane potential and [Ca^2+^]_i_ concentrations, respectively [11]. Positive controls included depolarising concentrations of KCl (30 mM) and alanine (10 mM) [14,26]. In 96-well plates, BRIN-BD11 cells were seeded and allowed to attach overnight in a CO_2_ incubator at 37 °C. The cells were then pre-incubated with 5.6 mM glucose KRB buffer for 10 min at 37 °C after the media was removed [27,28]. The cells were then treated with the FLIPR membrane potential or calcium dye for 60 min. A Flex Station 3 microplate reader was used to assess changes in signal intensity at wavelengths of 530 nm, 565 nm, and 550 nm for membrane potential and 485 nm, 525 nm, and 515 nm for intracellular calcium, accordingly [29].

### 2.4. Glycation of Insulin

The effects of EEAS on insulin glycation were investigated as previously reported, using aminoguanidine as a positive control [14]. Human insulin (1 mg/mL) and NaBH_3_CN (85.3 mg/mL) were incubated with D-glucose (246.5 mM) in the absence (Control) and presence (Treatment) of EEAS. After 24 h, the reaction was stopped with 0.5 M acetic acid and glycated and non-glycated insulins were quantified by reversed-phase high-performance liquid chromatography (RP-HPLC) [30].

### 2.5. Cellular Glucose Uptake

Adipocytes derived from 3T3L1 fibroblast cells were treated with EEAS for 30 min at 37 °C in a CO_2_ incubator, with/without 100 nM insulin. Further incubations were performed with 2-NBDG, 2-(N-(7-Nitrobenz-2-oxa-1,3-diazol-4-yl)Amino)-2-Deoxyglucose (50 nM) for 5 min. After rinsing cells with ice-cold PBS, coverslips were fixed onto the slides. In order to measure the glucose uptake/insulin action, a fluorescence microscope was used to capture magnified images of the fluorescence intensity [14].

### 2.6. In Vitro DPP-IV Enzyme Activity

A fluorometric assay using the substrate Gly-Pro-AMC was performed to investigate the effects of EEAS on DPP-IV enzyme activity using a previously established protocol [30]. DPP-IV enzyme and substrate (Gly-Pro-AMC) concentrations of 8 mU/mL and 200 µM were used to assess enzyme activity plus (Treatment)/minus (Control) EEAS in 96-well microplates (Greiner Bio-One Ltd., Gloucestershire, UK). The fluorescence intensity was measured with a Flex Station 3 (Molecular Devices, Sunnyvale, CA, USA) equipped with a 2.5 nm slit width at excitation and emission wavelengths of 370 and 440 nm, respectively. The known DPP-IV inhibitor sitagliptin was used as a positive control for this assay.

### 2.7. Starch Digestion

Chronological incubations with heat-stable α-amylase and amyloglucosidase (Sigma-Aldrich, St. Louis, MO, USA) were performed to evaluate the effects of EEAS on starch digestion, as outlined before [14]. A mixture of 100 mg/50 mL starch solution plus/minus EEAS was incubated for 20 min at 80 °C with heat-stable α-amylase (0.01%). Additional incubation for 30 min at 60 °C with 0.1% amyloglucosidase (from *Rhizopus* sp.) was performed. The GOD/PAP (Randox GL 2623) method was used to analyse glucose from aliquots of samples that were afterwards stored at 4 °C. Acarbose was used as a positive control for this assay.

### 2.8. In Vitro Glucose Diffusion

The effects of EEAS on endogenous glucose diffusion were measured using a cellulose ester (CE) dialysis tube (20 cm × 7.5 mm, Spectra/Por^®^CE layer, MWCO: 2000, Spectrum, The Netherlands) which was filled with 2 mL 0.9% NaCl and 220 mM glucose with/without EEAS. The two ends of the tube were tightly sealed before inserting it in 45 mL of 0.9% NaCl solution. The tubes were placed on an orbital shaker at room temperature for 24 h. The GOD/PAP method (Randox GL 2623) was used to estimate the amount of glucose diffused into the external solution using 0.5 mL aliquots of the dialysate mixture [31].

### 2.9. Animals

Sprague Dawley male rats (6–8 weeks old, Harlan Ltd., Blackthorn, UK) weighing between 150 and 200 g were fed a high-fat diet (45% fat, 20% protein, 35% carbohydrate, equivalent to 26.15 KJ/g total energy, Special Diet Service, Essex, UK) for six to eight weeks. Normal rats were fed a standard diet (10% fat, 30% protein, 60% carbohydrate, equivalent to 12.99 KJ/g total energy, Trouw Nutrition, Cheshire, UK). The animals were maintained at a specific temperature and humidity range (25 ± 0.5 °C and 65–70%, respectively) and using a 12 h automatic light on–off facility to ensure a day–night circadian rhythm. The effects of EEAS on residual gut sucrose content, intestinal glucose absorption, and gastrointestinal (GI) motility were assessed using a range of in vivo assays. An intraperitoneal dose of sodium pentobarbital solution (50 g/kg) was used to anesthetise the rats, and a midline abdominal incision was performed from about 1 cm below the xiphoid to the pelvis. The intestine was divided into five sections: the upper 20 cm, middle, and lower 20 cm of the small intestine, the caecum, and the large intestine. The Animal Welfare and Ethical Review Board (AWERB) at Ulster University approved all studies in May 2018 and they were carried out under the UK Home Office Animal project/personal licence numbers PIL1822 and PPL 2804, which were granted in May 2016 and February 2017, respectively. Both the UK Act 1986 and EU Directive 2010/63EU were followed in the execution of the experiments and it was ensured that no animals were harmed throughout study.

### 2.10. Oral Glucose Tolerance

The rats fed a high-fat diet were fasted overnight and given oral glucose (18 mmol/kg, body weight (b.w.)) alone (control) or in addition to EEAS (250 mg/5 mL/kg b.w.). Blood glucose levels were monitored using an Ascencia Contour Blood Glucose Meter (Bayer, Newbury, UK) at 0 (before oral gavage), 30, 60, 120, and 180 min following tail vein bleeding. Heparinised microvessel blood collection tubes (Sarstedt, Numbrecht, Germany) were used to collect the blood samples. Centrifugation at 12,000 rpm for 5 min at 4 °C separated the plasma from the blood, and the samples were kept at −20 °C for the insulin assay [32].

### 2.11. In Vivo DPP-IV Enzyme Activity

Fluorometric assays were used to assess the effects of EEAS on the DPP-IV enzyme levels in the plasma, as previously described [13]. Blood was obtained from the rats’ tail tips before or after the oral administration of EEAS (250 mg/5 mL/kg), DPP-IV inhibitors sitagliptin (10 mol/5 mL/kg) and vildagliptin (10 mol/5 mL/kg), or a saline control, as shown in Figure 3D. Active GLP-1 (7-36) levels in the plasma were measured using a GLP-1 (Active) ELISA Kit (EGLP-35K, Merck Millipore, Dorset, UK).

### 2.12. Residual Gut Sucrose Content

To examine the effects of EEAS on the absorption of sucrose from the GI tract, the residual sucrose content was measured as described earlier [33]. Rats fasted for 24 h were orally administered a sucrose solution (2.5 g/5 mL/kg) in the presence or absence of EEAS (250 mg/5 mL/kg and 500 mg/5 mL/kg). Blood samples were obtained from the tail tip at 0, 30, 60, 120, and 240 min. To determine the amount of unabsorbed sucrose, the rats were sacrificed at the specified times as above and the gastrointestinal tract (GIT) was divided into six segments as indicated in Figure 4C–H. Each segment was washed out with ice-cold saline, acidified with H_2_SO_4,_ and centrifuged at 3000 rpm (1000× *g*) for 10 min. The supernatant obtained was boiled for 2 h to hydrolyse the sucrose followed by neutralisation with sodium hydroxide to bring the pH to 7.0–7.4 [34]. The blood glucose levels after sucrose load and the amount of glucose liberated from the residual sucrose in the GIT were measured [35].

### 2.13. Intestinal Glucose Absorption

An in situ intestinal perfusion approach was used to explore the effects of EEAS on glucose absorption in the intestine. After 36 h of fasting, non-diabetic rats were anesthetised with 50 g/kg b.w. of sodium pentobarbital [36]. The EEAS (5 mg/mL and 10 mg/mL, equivalent to 0.25 g/5 mL/kg and 0.50 g/5 mL/kg, respectively) was diluted in a Krebs Ringer Bicarbonate (KRB) buffer solution supplemented with glucose and loaded through the rats’ pylorus. The perfusate was collected via a catheter placed at the end of the ileum. The control animals received KRB buffer with glucose only. The intestinal perfusion was conducted at a steady rate of 0.5 mL/min for 30 min at 37 °C. The amount of glucose in the solution prior to and following the perfusion was measured to find out the percentage of glucose absorbed [13,37].

### 2.14. Gastrointestinal Motility

An evaluation of gastrointestinal motility was carried out using BaSO_4_ milk as per Chatterjee’s method [38]. The milk was prepared by adding 10% *w*/*v* BaSO_4_ to a suspension of 0.5% carboxymethyl cellulose. Fasted (12 h) non-diabetic rats were orally administered with EEAS (250 mg/kg b.w.), while the control group was treated with distilled water only (10 mL/kg). The animals were fed BaSO_4_ milk by oral gavage 1 h after EEAS administration, and 15 min afterwards, the animals from both groups were sacrificed. The distance travelled by the BaSO_4_ milk throughout the entire length of the small intestine (from the pylorus to the ileo–caecal junction) was measured to calculate the percentage distance [11]. The standard drug glibenclamide (5 mg/5 mL/kg) was used as positive control.

### 2.15. Feeding Test

The feeding test was performed using animals fasted for 12 h before the start of the experiment. The food intake was measured at specific times before or after the oral administration of water, EEAS (250 mg and 500 mg/5 mL/kg), or the standard drug glibenclamide as a positive control (100 mg/5 mL/kg), as indicated in Figure 5D.

### 2.16. Crude Extract Purification

RP-HPLC was used to analyse the EEAS crude extract diluted in 0.12% (*v*/*v*) TFA/water. The filtered extract was loaded on a Vydac 218TP1022 preparative stainless steel 10 μm C-18 column (22 × 250 mm) (Grace, Deerfield, IL, USA), equilibrated with 0.12% (*v*/*v*) TFA/water at a flow rate of 5 mL/min. Acetonitrile was used as an eluent using linear gradients of 20% over 10 min and 70% over 40 min. Individual peak retention times were recorded and peak fractions detected at 254 nm were collected [14]. Insulinotropic peak fractions were subsequently assessed using a semi-preparative Vydac 208TP510 (10 × 250 mm) 5 μm C-18 column (Phenomenex, UK) eluting with acetonitrile as indicated above, at flow rate of 1 mL/min.

### 2.17. Mass Spectrometry Analysis

Liquid chromatography–electrospray ionisation mass spectrometry (LC-ESI-MS) was used to analyse the molecular weights of the peak fractions of EEAS. These were separated using a Kinetex F5 LC column (150 × 4.6 mm; 5 µm) (Phenomenex, UK) with UV detection at 220–256 nm on a Spectra System LC (Thermo Separation Products) as previously reported [30].

### 2.18. Statistical Analysis

GraphPad Prism 5 was used for the data analysis and interpretation. The data were analysed using an unpaired Student’s *t*-test (two-tailed *p*-values) and one-way ANOVA with Bonferroni post hoc tests. The values are reported as mean ± SEM, with *p* < 0.05 denoting the significance limit.

## 3. Results

### 3.1. EEAS and Insulin Release from BRIN-BD11 Cells

**Figure 1:**
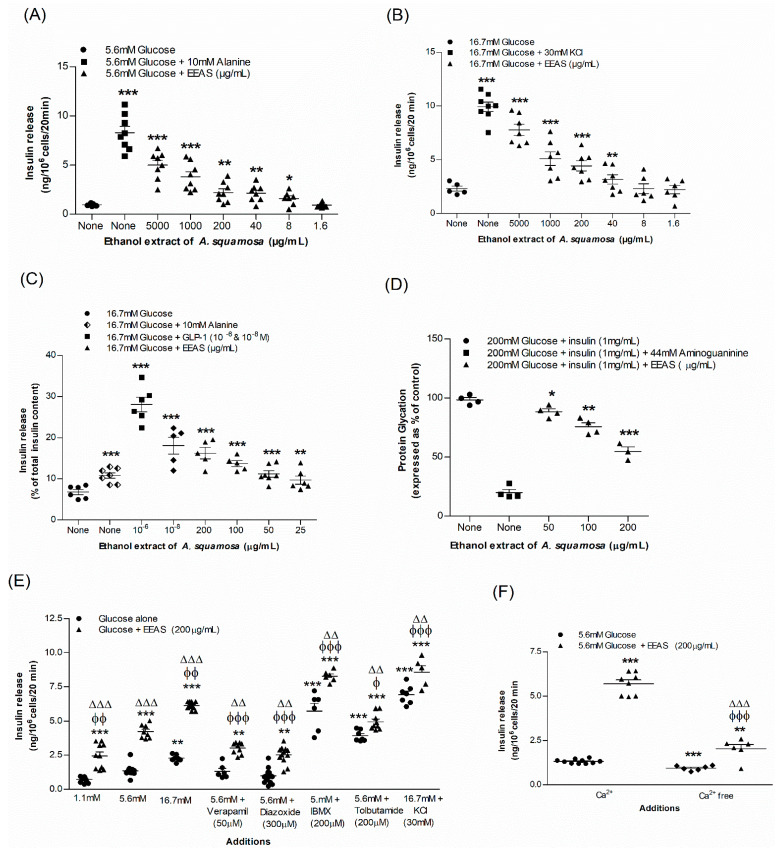
Effects of EEAS on insulin secretion from (**A**,**B**) clonal pancreatic β-cells (BRIN-BD11) and (**C**) islets of Langerhans, (**D**) glycation of protein, (**E**) secretion of insulin with known stimulators/inhibitors, and (**F**) with/without extracellular calcium from clonal β-cells. Values *n* = 4–8 for insulin secretion and glycation of protein are mean ± SEM. *, **, *** *p* < 0.05–0.001 compared to control. ϕ, ϕϕ, ϕϕϕ *p* < 0.05–0.001 compared to 5.6 mM glucose with EEAS. ΔΔ, ΔΔΔ *p* < 0.05–0.001 compared to respective incubation without EEAS. EEAS, ethanol extract of *A. squamosa*.

The ethanol extract of *A. squamosa* (EEAS) induced insulin secretion from BRIN-BD11 cells in a concentration-dependent manner (1.6–5000 µg/mL) (*p* < 0.05–0.001; Figure 1A,B). Insulin secretion in the presence of EEAS was increased by 2–6.5-fold compared to glucose concentrations of 5.6 and 16.7 mM alone (*p* < 0.05–0.001; Figure 1A,B). The positive controls, alanine (10 mM), and KCl (30 mM) intensified the insulin secretion significantly (*p* < 0.001; Figure 1A,B). EEAS concentrations of up to 200 µg/mL had no effect on the cell efficacy, but doses above 200 µg/mL increased LDH release by 20% to 75% (data not shown).

EEAS showed a concentration-dependent (50–200 μg/mL) increase in glucose-induced insulin secretion by 1.4–2.8-fold compared to 16.7 mM glucose alone (*p* < 0.01–0.001). The positive controls GLP-1 (10^−6^ and 10^−8^ M) and alanine (10 mM) also significantly increased insulin release (*p* < 0.001). The incretin hormone GLP-1 (10^−6^ and 10^−8^ M)-induced insulin release from mouse islets was more potent than that of alanine (Figure 1C).

The insulin modulators, glucose (16.7 mM), isobutylmethylxanthine (IBMX; 200 µM), and tolbutamide (200 µM) further stimulated the insulin-releasing ability of EEAS (*p* < 0.01–0.00; Figure 1E) by 2.7, 1.5 and 1.3-fold, respectively. At 16.7 mM glucose, EEAS maintained the regulation of insulin secretion from cells depolarised with 30 mM KCl (*p* < 0.01; Figure 1E). On the other hand, the insulin-releasing inhibitors diazoxide (50 µM) and verapamil (200 µM), as well as Ca^2+^-free conditions, led to a decrease in the insulin release from clonal pancreatic BRIN BD11 β-cells, although this was not completely abolished (*p* < 0.01; Figure 1E,F). 

### 3.2. EEAS and Glycation of Insulin

The known inhibitor aminoguanidine (44 mM) reduced insulin glycation by 80.5% (*p* < 0.001). In the presence of EEAS (50–200 µg/mL), the percentage of insulin glycation was attenuated by 8–46% (*p* < 0.05–0.001; Figure 1D).

### 3.3. EEAS and Membrane Depolarisation and [Ca^2^^+^]_i_

**Figure 2:**
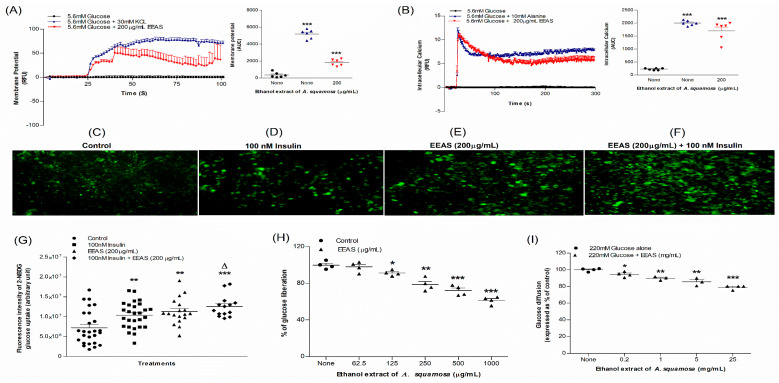
Effects of EEAS on (**A**) membrane potential and (**B**) intracellular calcium in clonal pancreatic β cells (BRIN BD11), and (**C**–**G**) glucose uptake, (**H**) starch digestion, and (**I**) glucose diffusion in vitro. Changes of fluorescence intensity in differentiated 3T3L1 adipocytes incubated with EEAS (**E**) without or (**F**) with 100 nM insulin. Values *n* = 6 for membrane potential and intracellular calcium, *n* = 4 for uptake of glucose, digestion of starch, and diffusion of glucose are mean ± SEM.*, **, *** *p* < 0.05–0.001 compared to control. ^Δ^ *p* < 0.05 compared to 100 nM insulin alone.

In the presence of 5.6 mM glucose, EEAS depolarised (*p* < 0.001) BRIN BD11 cells and increased (*p* < 0.001) the intracellular calcium [Ca^2+^]_i_ concentrations (Figure 2A,B). The positive controls KCl (30 mM) and alanine (10 mM) induced membrane depolarisation (*p* < 0.001) and increased intracellular calcium levels (*p* < 0.001; Figure 2A,B). 

### 3.4. EEAS and Glucose Uptake and Insulin Action

Adipocyte cells (3T3L1) were used to assess the effects of EEAS on insulin action using the glucose analogue 2-NBDG fluorescent hexose. Figure 2C–F depict the microscopic fluorescence intensity of 2-NBDG uptake. In 3T3L1-differentiated adipocyte cells, EEAS (200 μg/mL) improved the insulin action two-fold (*p* < 0.05–0.01), while with/without insulin (100 nM), EEAS increased glucose uptake by 33% and 22%, respectively (*p* < 0.01; Figure 2G). 

### 3.5. EEAS and Starch Digestion

Figure 2H demonstrates the impact of EEAS on starch digestion. EEAS at 125–1000 µg/mL suppressed enzymatic activity by 10–39% (*p* < 0.05–0.001: Figure 2H). The standard inhibitory agent, acarbose (1 mg/mL), reduced starch digestion by 87% (data not shown).

### 3.6. EEAS and Glucose Diffusion In Vitro

Figure 2I depicts the inhibition of glucose diffusion and absorption in the presence of EEAS after 24 h incubation. At a high concentration (25 mg/mL), EEAS showed 21% inhibition (*p* < 0.001; Figure 2I). At a low concentration (0.2 mg/mL), the percentage of inhibition decreased to 5.5% (*p* < 0.05; Figure 2I).

### 3.7. EEAS and In Vitro DPP-IV Enzymatic Activity

**Figure 3:**
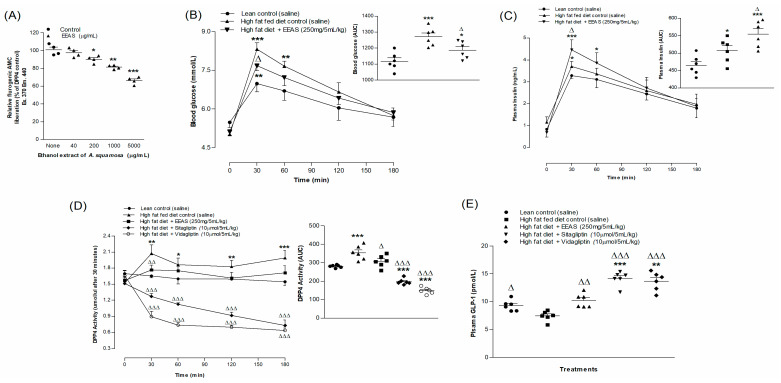
Effects of EEAS on (**A**) DPP-IV enzyme in vitro, (**B**) glucose tolerance, (**C**) plasma insulin, (**D**) DPP-IV, and (**E**) active GLP-1 (7-36) in high-fat-fed rats. In vivo parameters were evaluated before and after oral gavage of glucose alone (18 mmol/kg body weight (b.w.), control) or with EEAS (250 mg/5 mL/kg b.w.), sitagliptin and vidagliptin (both at 10 μmol/5 mL/kg b.w.). Plasma active GLP-1 (7-36) levels was assayed at 30 min after treatments. Values *n* = 4 for in vitro DPP-IV enzyme activity and *n* = 6 for in vivo parameters are mean ± SEM. *, **, *** *p* < 0.05–0.001 compared to control. ^Δ, ΔΔ, ΔΔΔ^ *p* < 0.05–0.001 compared to high-fat-fed diet control rats.

The activity of the dipeptidyl peptidase-IV enzyme in the presence of various concentrations of EEAS is illustrated in Figure 3A. At concentrations ranging from 200 to 5000 µg/mL, EEAS produced a 9–33% (*p* < 0.05–0.001) decrease in AMC liberation from Gly-Pro-AMC in the presence of the DPP-IV enzyme. The known DPP-IV inhibitor sitagliptin suppressed AMC liberation from Gly-Pro-AMC by 96% (data not shown).

### 3.8. EEAS and Oral Glucose Tolerance and Plasma Insulin, DPP-IV and Active GLP-1 (7-36) Levels

The oral gavage of glucose (18 mmol/5 mL/kg b.w.) with EEAS improved oral glucose tolerance at 30 and 60 min in HFF rats (*p* < 0.05; Figure 3B). This was accompanied with increased plasma insulin levels at 30 min (*p* < 0.05; Figure 3C). EEAS demonstrated a 15% decrease (*p* < 0.01) in DPP-IV enzyme activity, while this decreased to 63–68% (*p* < 0.001; Figure 3D) with sitagliptin and vidagliptin, respectively. The oral administration of EEAS (at 30 min) elevated the active GLP-1 (7-36) concentrations in plasma by 37% (*p* < 0.01; Figure 3E). This was increased by 84–90% (*p* < 0.001) with sitagliptin and vidagliptin (Figure 3E).

### 3.9. EEAS and Blood Glucose after Sucrose Load

**Figure 4:**
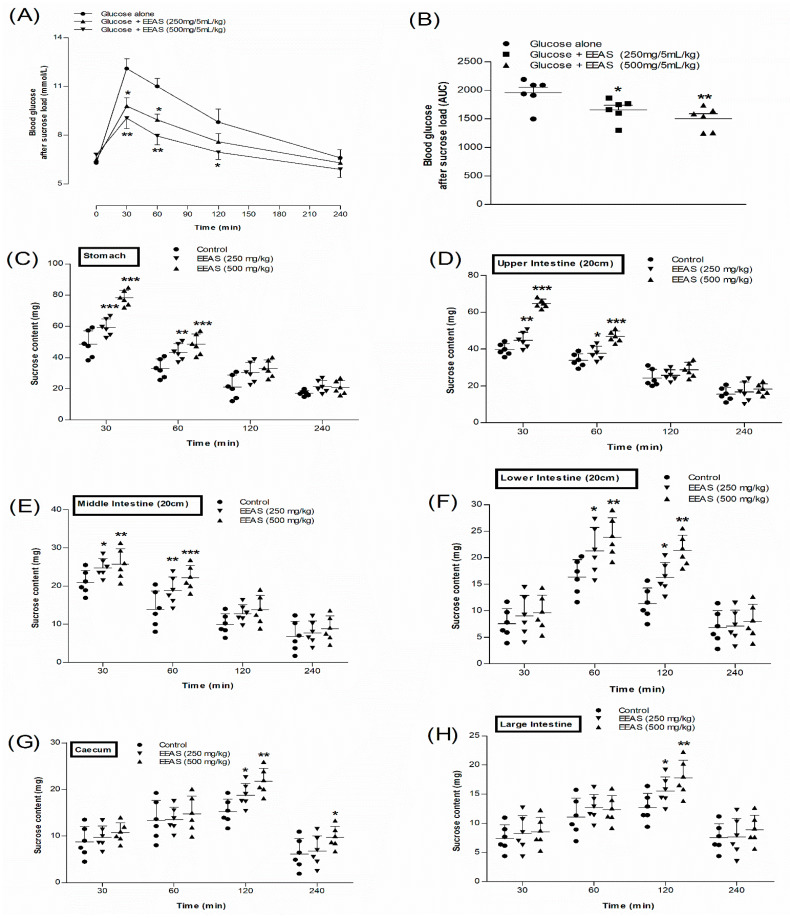
Effects of EEAS on (**A**,**B**) blood glucose after sucrose load and gastrointestinal sucrose content in (**C**) stomach, (**D**) upper, (**E**) middle, and (**F**) lower intestine, (**G**) caecum, and (**H**) large intestine after oral sucrose loading in non-diabetic rats. Rats were fasted for 20 hr prior to oral administration of a sucrose solution (2.5 g/5 mL/kg b.w.) with (treated group) or without (control group) EEAS (250 mg/5 mL/kg and 500 mg/5 mL/kg b.w.). Values *n* = 6 for gut sucrose content and blood glucose after sucrose load are mean ± SEM. *, **, *** *p* < 0.05–0.001 compared to control.

EEAS (250 mg/5 mL/kg b.w.) showed significant (*p* < 0.05) reduction in serum glucose levels at 30 and 60 min (Figure 4A) compared to the control (sucrose alone). However, at a higher dose (500 mg/5 mL/kg), EEAS decreased the serum glucose levels at 30, 60, and 120 min (*p* < 0.05–0.01; Figure 4A). There was no significant decrease in the glucose levels in rats receiving oral sucrose alone (Figure 4A). The area under the curve (AUC) indicated that the blood glucose levels were reduced by 15–23% in the presence of EEAS compared to the control (Figure 4B).

### 3.10. EEAS and Residual Gut Sucrose Content

Figure 4C–H indicate the impact of oral sucrose administration (2.5 g/5 mL/kg b.w.) on the residual gut sucrose content in normal rats plus/minus EEAS. Sucrose absorption was reduced with EEAS (250 and 500 mg/5 mL/kg b.w.) in the stomach, upper, middle, and lower small intestine at 30 and 60 min, respectively (*p* < 0.05–0.001; Figure 4C–H). Some sucrose remained unabsorbed in the lower intestine, caecum, and large intestine of the treated groups after 2 h (*p* < 0.05–0.01; Figure 4F–H). The sucrose content was almost nil after 4 h in the control group (sucrose alone), whereas a small amount of sucrose was detected in the caecum of the group treated with EEAS at a dose of 500 mg/5 mL/kg b.w. (*p* < 0.05; Figure 4G).

### 3.11. EEAS and Intestinal Gut Perfusion In Situ

**Figure 5:**
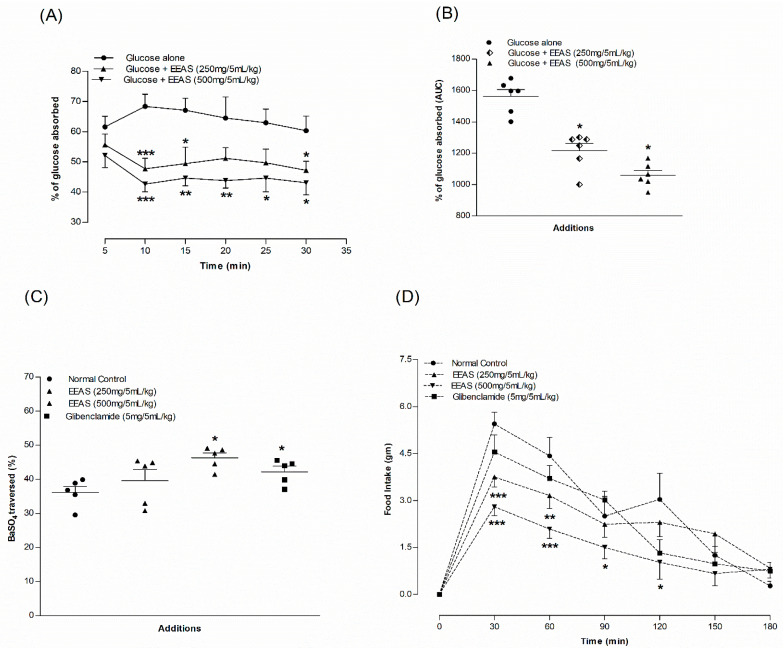
Effects of EEAS on (**A**) intestinal glucose absorption expressed as line graph and (**B**) area under the curve, (**C**) gut motility and (**D**) feeding test. Rats were fasted for 36 h and the intestine was perfused with glucose (54 g/L) with (treated group) or without (control group) ethanol extract of *A. squamosa* leaves (5 mg/mL and 10 mg/mL; each subject received 15 mL of perfusion). Values *n* = 6 for gut perfusion and motility and feeding test are mean ± SEM. *, **, *** *p* < 0.05–0.001 compared to control.

A combination of EEAS (500 mg/5 mL/kg b.w.) with glucose caused a significant decrease in intestinal glucose absorption (*p* < 0.05–0.001; Figure 5A) throughout most of the perfusion time. Similarly, in the presence of EEAS at 250 mg/5 mL/kg, glucose absorption was suppressed in the gut at 10, 15, and 30 min compared to the control (glucose alone) *(p* < 0.05–0.001; Figure 5A). The area under the curve indicated that EEAS suppressed intestinal glucose absorption by 22–32% (Figure 5B).

### 3.12. EEAS and Gut Motility

EEAS (500 mg/5 mL/kg b.w.) increased gastrointestinal motility by 11% compared to the control (*p* < 0.05); however, there was no significant change at 250 mg/5 mL/kg (Figure 5C). Sulfonylureas and glibenclamide (5 mg/5 mL/kg) also improved gut motility by 6.5% (*p* < 0.05; Figure 5C).

### 3.13. EEAS and Feeding Test

EEAS (500 mg/5 mL/kg) improved food intake at 30, 60, 90, and 120 min (*p* < 0.05–0.001; Figure 5D). With a dose of 250 mg/5 mL/kg, this improvement was only observed at 30 and 60 min (*p* < 0.01–0.001; Figure 5D). The positive control glibenclamide (5 mg/5 mL/kg) also decreased food intake, but not significantly compared to the control.

### 3.14. Extract Purification and Analysis

**Figure 6:**
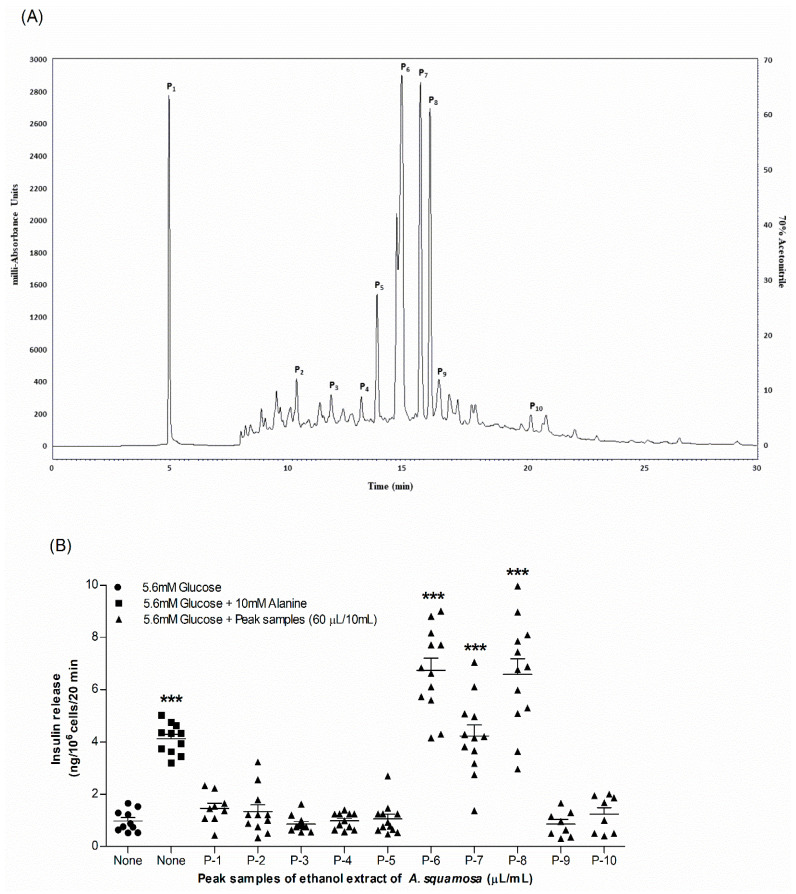
Representative (**A**) HPLC profile and (**B**) insulinotropic properties of EEAS peak fractions (1–10). At a flow rate of 5.0 and 1.0 mL/min, samples were run in RP-HPLC using linear gradients of 20% over 10 min and 70% over 40 min. Peak fractions (1–10) detected at 254 nm were further tested for insulin-releasing properties using clonal pancreatic BRIN-BD11 β-cells. Values *n* = 8 for insulin release are mean ± SEM. *** *p* < 0.001 compared to control.

**Figure 7:**
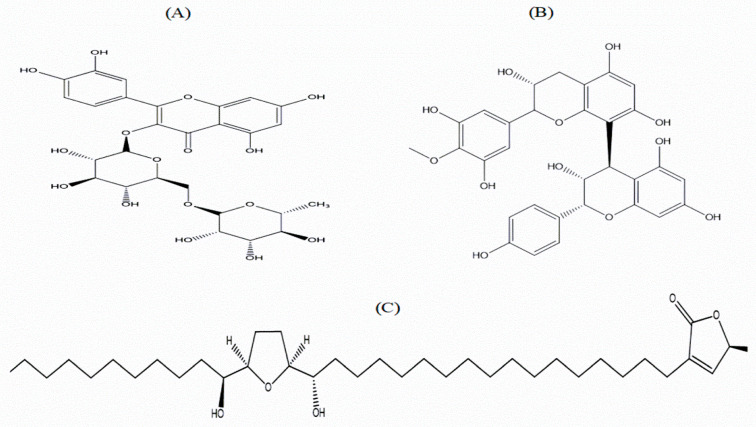
Chemical structure of putative phytomolecules derived from the EEAS. (**A**) Rutin, (**B**) proanthocyanidin, and (**C**) squafosacin G chemical structures with their respective molecular formulae: C_27_H_30_O_16_, C_31_H_28_O_12_, and C_37_H_68_O_5_.

The chemical analysis of EEAS was performed using RP-HPLC (Table 1; Figure 6A). The peak fractions (P-1 to P-10) collected were further assessed for insulinotropic properties from clonal pancreatic BRIN BD11 β-cells (Figure 6B). Insulin secretion was significantly induced by peak fractions P-6, P-7, and P-8 (*p* < 0.001; Figure 6B). However, cell toxicity was attributed to P-2, P-5, and P-7. The standard control alanine (10 mM) increased insulin secretion significantly (*p* < 0.001; Figure 6B). All peak fractions of interest were subsequently analysed via LC-MS (Table 1). Peak fractions P-5, P-6, P-7, and P-8 exhibited molecular masses of 741.4, 609.3, 592.3, and 592.0 Da, respectively. The chemical structures of the putative bioactive phytoconstituents in EEAS are illustrated in Figure 7A–C.

## 4. Discussion

*Annona squamosa*, commonly known as “Ata” in the South Asian subcontinent, has been found to exert promising antidiabetic activity [39,40,41], although information regarding its mode of action requires further investigations. This study evaluated the insulinotropic and antidiabetic properties of the ethanol extract of *A. squamosa* leaves in vitro on clonal pancreatic BRIN BD11 β-cells, isolated mouse islets, and other in vitro parameters such as starch digestion, insulin glycation, glucose diffusion, glucose uptake, and DPP-IV activity as well as in vivo using rat models. EEAS induced a concentration-dependent increase in insulin release from clonal pancreatic BRIN BD11 β-cells. This increase in insulin release in the presence of EEAS was also observed from glucose-stimulated isolated mouse islets.

Mechanistic studies in the presence of insulin releasing/inhibiting modulators were carried out to investigate the effect of EEAS on distinct signal transduction pathways involved in insulin secretion. In the presence of diazoxide (K_ATP_ channel opener) and verapamil (voltage-dependent Ca^2+^ channel blocker), the insulin-releasing effect of EEAS was reduced. The sulfonylurea tolbutamide stimulates insulin release by depolarising the β cell membrane via inhibiting K_ATP_ channels, activating voltage-dependent Ca^2+^ channels and triggering intracellular Ca^2+^ influx [42]. In the presence of tolbutamide, the insulin-releasing effect of EEAS was increased. These findings suggest that at least part of the insulin-stimulating activity of EEAS is mediated by the K_ATP_ and Ca^2+^ ion channel-independent pathways with the possible involvement of intracellular messenger pathways such as the adenylate cyclase/cAMP or phosphatidylinositol (PI_3_) pathway, or direct impact on exocytosis [30]. IBMX, a cAMP phosphodiesterase inhibitor, also significantly improved insulin secretion with EEAS, which is consistent with the above results. Interestingly, *A. squamosa* is traditionally used to treat asthma [43] due to its ability to increase cAMP in bronchial smooth muscles, thereby causing a relaxation of the airways and a decrease in smooth muscle cellular proliferation [44]. 

EEAS was further investigated for its effects on glucose uptake using 3T3L1 adipocyte cells. EEAS significantly enhanced the insulin action, which promotes signal transduction in target cells and increases glucose uptake and storage [45]. The activation of insulin receptors leads to internal cellular mechanisms that directly affect glucose uptake. These mechanisms regulate postprandial glucose in extra-pancreatic cells, such as adipocytes and skeletal muscle cells, by enhancing the translocation of the glucose transporter GLUT4. In response to glucose, insulin activates the PI3/Akt pathway, which stimulates the protein phosphorylation cascade and causes an increase in glucose uptake [46]. Further in-depth studies are required to ascertain the exact contribution of EEAS in this mode of action.

Insulin glycation is a key post-translational alteration in the pathogenesis of diabetes that can lead to long-term problems [47]. The glycation of proteins with short half-lives, such as insulin, can lead to the formation of advanced glycation end products (AGEs), resulting in insulin resistance [48,49,50]. In this study, EEAS was found to decrease insulin glycation in vitro, indicating its potential against insulin resistance and diabetes-associated complications. Moreover, recent studies demonstrated that *A. squamosa* has antioxidant properties that may decrease oxidative stress via the inhibition of lipid peroxidation in pancreatic β-cells [27,39].

In vitro studies were also conducted to examine the effects of EEAS on carbohydrate digestion. The α-glucosidase inhibitor acarbose used as a positive control was found to completely terminate glucose liberation by inhibiting carbohydrate digestion. EEAS also suppressed carbohydrate digestion and reduced glucose absorption. It has been previously observed that *A. squamosa* could affect carbohydrate digestion by inhibiting the activity of the α-glucosidase and α-amylase enzymes [51]. Further studies on glucose diffusion revealed that EEAS decreases gastrointestinal glucose absorption and diffusion. These results are consistent with previous findings reporting that the hot water extract of *A. squamosa* could suppress glucose absorption in both alloxan and streptozotocin (STZ)-induced diabetic rats [52].

In vitro studies with EEAS showed promising effects, which further instigated the performance of in vivo studies using an HFF rat model showing obesity and insulin resistance. EEAS improved oral glucose tolerance and plasma insulin in HFF rats and food intake in non-diabetic rats. These findings corroborate with the results of in vitro studies of *A. squamosa*. Recent studies reported that phytoconstituents including rutin, quercitrin, and proanthocyanidin also have insulin secretory and glucose-lowering properties [14,30,53].

In recent times, there has been a growing interest in DPP-IV inhibitors—a class of oral anti-hyperglycaemic drugs that lower blood glucose levels—as a potential treatment for T2DM [54]. The DPP-IV enzyme is responsible for the rapid degradation of the incretin hormones GLP-1 and GIP under normal physiological conditions by cleaving in their N-terminal residues to form GLP-1 (9-36) and GIP (3-42) [55,56]. The inhibition of DPP-IV delays the breakdown of the incretin hormones and positively modulates insulin secretion [57]. Therefore, DPP-IV enzyme inhibition and the development of the glucagon-like peptide-1 receptor (GLP-1R) agonists, resistant to enzymatic degradation, are important strategies for treating T2DM [56]. Our in vitro studies demonstrated the significant suppression of DPP-IV enzyme activity in a concentration-dependent manner after treatment with EEAS. Similarly, in HFF rats, EEAS significantly inhibited plasma DPP-IV enzyme activity and increased the active GLP-1 (7-36) levels in circulation. These results are in agreement with previous studies conducted on various medicinal plants with DPP-IV-inhibitory effects [22,58]. Earlier studies showed that phytochemicals from natural sources including *A. squamosa*, such as proanthocyanidin and rutin, have the ability to suppress DPP-IV enzyme activity [59,60].

EEAS significantly reduced glucose absorption during gut perfusion, which was assessed by determining the remaining amount of unabsorbed sucrose content in different segments of the gut. Treatment with EEAS caused significantly higher amounts of residual sucrose in the stomach and upper, middle, and lower intestine compared to the non-treated rats. EEAS also inhibited glucose absorption in the last three segments of the GI tract, which are important for nutritional absorption, including glucose [61]. It is known that, due to the lack of carriers in the GI tract, disaccharides are not normally absorbed [62]. Therefore, disaccharides must be broken down into monosaccharides prior to absorption in the GI tract. Thus, the increased sucrose content in the GI tract seen following EEAS administration may be attributable to a decrease in sucrose catabolism and digestion throughout the GI tract. Previous studies with *A. squamosa* and one of its phytochemicals, rutin, also showed a reduction in intestinal glucose absorption in STZ-induced diabetic rats [54,63]. We also observed that EEAS promotes gut motility in the BaSO_4_ milk study. This action may shorten the time remaining for carbohydrate absorption. As a consequence, glucose absorption in the circulation may decrease, causing a fall in blood glucose levels. These actions may be mediated by dietary fibres, which can influence the viscosity and transport time of the food contents in the GI tract [64]. The ability of *A. squamosa* to decrease glucose absorption and increase gut motility may be due to its high dietary fibre content [65].

The potential bioactive phytoconstituents of EEAS were investigated using RP-HPLC and LC-MS. Our preliminary screening led to the isolation of several peak fractions, including P-6, P-7, and P-8, which increased the insulin release from clonal pancreatic BRIN BD11 β-cells, and may be the phytoconstituents contributing to the antidiabetic activity of EEAS. Peak fractions P6, P-7, and P-8 possessed molecular masses corresponding to rutin, squafosacin G, and proanthocyanidin, respectively. These findings are consistent with prior studies of *A. squamosa* having rutin, squafosacin G, and proanthocyanidin [66,67,68]. Previous research on rutin and proanthocyanidin showed that these compounds could improve glucose tolerance and inhibit glucose absorption by inhibiting intestinal α-glucosidase enzyme activity [69,70]. Rutin and proanthocyanidin have also shown antioxidant properties in STZ-induced diabetic rats, supressing the production of AGEs via downregulating inflammatory factors, and protecting pancreatic β-cells from damage [71,72]. Squafosacin G is classified as an acetogenin [73] and these compounds are known as potent antioxidants due to their free radical-scavenging properties [74]. This indicates that squafosacin G may reduce oxidative stress and improve diabetes complications. Additional studies are warranted to fully confirm the structures of the aforementioned phytomolecules.

## 5. Conclusions

This study has demonstrated that the ethanol extract of *A. squamosa* leaves (EEAS) possesses antidiabetic activity by enhancing insulin secretion both in vitro in clonal pancreatic BRIN BD11 β-cells and in isolated mouse islets, as well as in vivo in HFF obese rats. Along with this, EEAS inhibited protein glycation, starch digestion, glucose diffusion, and DPP-IV enzyme activity and enhanced the active GLP-1 levels in circulation. Interestingly, the results of this study also revealed that EEAS improves glucose homeostasis by lowering glucose absorption in the gut, indicating that it may inhibit disaccharidase enzyme activity in rats. The presence of phytoconstituents such as rutin and proanthocyanidin may be a contributing factor to the insulin-releasing and glucose-lowering benefits of EEAS. Overall, studies carried out to date on *A. squamosa* support, to some extent, its traditional use against diabetes. Additional in-depth studies are needed to fully understand the exact nature of its bioactive phytoconstituents and exploit their possible use for T2DM in humans.

## Figures and Tables

**Table 1:** Molecular mass of peak samples of EEAS leaves obtained from the preparative RP-HPLC via LC-MS analysis.

Peak Samples	Retention Time (Min)	Theoretical Molecular Weight (Da)	Found Molecular Weight (Da)	Predicted Compounds
P_1_	5	-	-	Not determined
P_2_	10.3	-	-	Not determined
P_3_	11.8	-	-	Not determined
P_4_	13	-	-	Not determined
P_5_	14	-	741.4	Unknown
P_6_	15	610.5	609.3	Rutin
P_7_	15.6	592.9	592.3	Squafosacin G
P_8_	16	592.5	592.0	Proanthocyanidin
P_9_	16.5	-	-	Not determined
P_10_	20	-	-	Not determined

Peaks were separated on a Spectra System LC using a Kinetex F5 LC column (150 × 4.6 mm, 5 µm) (Phenomenex, UK). The mass-to-charge ratio (*m*/*z*) versus peak intensity was determined. P-5, P-6, P-7, and P-8 eluting at 14, 15, 15.6, and 16.5 min showed molecular ions at *m*/*z* 741.4, 609.3, 592.3, and 592.0 Da, respectively.

## Data Availability

The data are not available to the public due to some restrictions. However, on request, the corresponding author can share the data reported in this study.

## Abbreviations

2-NBDG	2-(N-(7-Nitrobenz-2-oxa-1,3-diazol-4-yl)Amino)-2-Deoxyglucose
AGE	Advanced glycation end products
AWERB	Animal Welfare and Ethical Review Board
BaSO_4_	Barium sulphate
cAMP	Cyclic adenosine monophosphate
CVD	Cardiovascular diseases
DPP-IV	Dipeptidyl peptidase IV
EEAS	Ethanol extract of *Annona squamosa*
FLIPR	Fluorometric imaging plate reader
GIP	Glucose-dependent insulinotropic polypeptide
GLP-1	Glucagon-like peptide-1
GLP-1R	Glucagon-like peptide-1 receptor
GLUT4	Glucose transporter type 4
HFF	High-fat-fed
IBMX	3-isobutyl-1-methylxanthine
IDF	International Diabetes Federation
KCl	Potassium chloride
LC-ESI-MS	Liquid chromatography–electrospray ionisation mass spectrometry
PI_3_	Phosphatidylinositol
RP-HPLC	Reverse-phase high-performance liquid chromatography
SGLT-2	Sodium–glucose cotransporter-2
STZ	Streptozotocin
T2DM	Type 2 diabetes mellitus
UARC	University Ayurvedic Research Center

## References

[1] Bastaki S. (2005). Diabetes mellitus and its treatment. Int. J. Diabetes Metab..

[2] Sun H., Saeedi P., Karuranga S., Pinkepank M., Ogurtsova K., Duncan B.B., Stein C., Basit A., Chan J.C., Mbanya J.C. (2022). IDF Diabetes Atlas: Global, regional and country-level diabetes prevalence estimates for 2021 and projections for 2045. Diabetes Res. Clin. Pract..

[3] Katsarou A., Gudbjörnsdottir S., Rawshani A., Dabelea D., Bonifacio E., Anderson B.J., Jacobsen L.M., Schatz D.A., Lernmark Å. (2017). Type 1 diabetes mellitus. Nat. Rev. Dis. Primers.

[4] DeFronzo R.A., Ferrannini E., Groop L., Henry R.R., Herman W.H., Holst J.J., Hu F.B., Kahn C.R., Raz I., Shulman G.I. (2015). Type 2 diabetes mellitus. Nat. Rev. Dis. Primers.

[5] Galicia-Garcia U., Benito-Vicente A., Jebari S., Larrea-Sebal A., Siddiqi H., Uribe K.B., Ostolaza H., Martín C. (2020). Pathophysiology of Type 2 Diabetes Mellitus. Int. J. Mol. Sci..

[6] Johnson M.L., Distelmaier K., Lanza I.R., Irving B.A., Robinson M.M., Konopka A.R., Shulman G.I., Nair K.S. (2016). Mechanism by Which Caloric Restriction Improves Insulin Sensitivity in Sedentary Obese Adults. Diabetes.

[7] Bhupathiraju S.N., Hu F.B. (2016). Epidemiology of obesity and diabetes and their cardiovascular complications. Circ. Res..

[8] Forbes J.M., Cooper M.E. (2013). Mechanisms of diabetic complications. Physiol. Rev..

[9] Alam U., Asghar O., Azmi S., Malik R.A. (2014). General aspects of diabetes mellitus. Handb. Clin. Neurol..

[10] Dyson P.A., Kelly T., Deakin T., Duncan A., Frost G., Harrison Z., Khatri D., Kunka D., McArdle P., Mellor D. (2011). Diabetes UK evidence-based nutrition guidelines for the prevention and management of diabetes. Diabet. Med..

[11] Ansari P., Azam S., Seidel V., Abdel-Wahab Y.H.A. (2022). In vitro and in vivo antihyperglycemic activity of the ethanol extract of *Heritiera fomes* bark and characterization of pharmacologically active phytomolecules. J. Pharm. Pharmacol..

[12] Bastin M., Andreelli F. (2019). Dual GIP–GLP1-receptor agonists in the treatment of type 2 diabetes: A short review on emerging data and therapeutic potential. Diabetes Metab. Syndr. Obes. Targets Ther..

[13] Ansari P., Azam S., Hannan J.M.A., Flatt P.R., Wahab Y.H.A. (2020). Anti-hyperglycaemic activity of *H. rosa-sinensis* leaves is partly mediated by inhibition of carbohydrate digestion and absorption, and enhancement of insulin secretion. J. Ethnopharmacol..

[14] Ansari P., Flatt P.R., Harriott P., Abdel-Wahab Y.H.A. (2020). Evaluation of the antidiabetic and Insulin Releasing Effects of *A. squamosa*, Including Isolation and Characterization of Active Phytochemicals. Plants.

[15] Ansari P., Flatt P.R., Harriott P., Abdel-Wahab Y.H.A. (2021). Anti-hyperglycaemic and insulin-releasing effects of *Camellia sinensis* leaves and isolation and characterisation of active compounds. Brit. J. Nutr..

[16] Ansari P., Flatt P.R., Harriott P., Hannan J.M.A., Abdel-Wahab Y.H.A. (2021). Identification of Multiple Pancreatic and Extra-Pancreatic Pathways Underlying the Glucose-Lowering Actions of *Acacia arabica* Bark in Type-2 Diabetes and Isolation of Active Phytoconstituents. Plants.

[17] Ansari P., Akther S., Hannan J.M.A., Seidel V., Nujat N.J., Abdel-Wahab Y.H.A. (2022). Pharmacologically Active Phytomolecules Isolated from Traditional Antidiabetic Plants and Their Therapeutic Role for the Management of Diabetes Mellitus. Molecules.

[18] Arumugam G., Manjula P., Paari N. (2013). A review: Anti diabetic medicinal plants used for diabetes mellitus. J. Acute Dis..

[19] Tran N., Pham B., Le L. (2020). Bioactive Compounds in Anti-Diabetic Plants: From Herbal Medicine to Modern Drug Discovery. Biology.

[20] Berglund L.M., Lyssenko V., Ladenvall C., Kotova O., Edsfeldt A., Pilgaard K., Alkayyali S., Brøns C., Forsblom C., Jonsson A. (2015). Glucose-Dependent Insulinotropic Polypeptide Stimulates Osteopontin Expression in the Vasculature via Endothelin-1 and CREB. Diabetes.

[21] Flatt P.R. (2008). Dipeptidyl Peptidase IV (DPP IV) and Related Molecules in Type 2 Diabetes. Front. Biosci..

[22] Pandey K., Sinha A., Perween Z. (2020). Important medicinal plants with their medicinal uses from Jharkhand State. Int. J. Res. Eng. Sci. Manag..

[23] Padmanabhan P., Paliyath G. (2016). Annonaceous Fruits. Encyclopedia of Food and Health.

[24] Bhat R., Paliyath G. (2016). Fruits of Tropical Climates: Dietary Importance and Health Benefits. Encyclopedia of Food and Health.

[25] Bhattacharya A., Chakraverty R. (2016). The pharmacological properties of *Annona squamosa* Linn: A Review. Int. J. Pharm. Eng..

[26] Pandey N., Barve D. (2011). Phytochemical and pharmacological review on *Annona squamosa* Linn. Int. J. Res. Pharm. Biomed. Sci..

[27] Kaleem M., Asif M., Ahmed Q., Bano B. (2006). Antidiabetic and antioxidant activity of *Annona squamosa* extract in streptozotocin-induced diabetic rats. Singap. Med. J..

[28] Hannan J.M.A., Ansari P., Azam S., Flatt P.R., Wahab Y.H.A. (2020). Effects of *Spirulina platensis* on insulin secretion, dipeptidyl peptidase IV activity and both carbohydrate digestion and absorption indicate potential as an adjunctive therapy for diabetes. Brit. J. Nutr..

[29] Ansari P., Flatt P.R., Harriott P., Abdel-Wahab Y.H.A. (2021). Insulinotropic and antidiabetic properties of *Eucalyptus citriodora* leaves and isolation of bioactive phytomolecules. J. Pharm. Pharmacol..

[30] Ansari P., Flatt P.R., Harriott P., Abdel-Wahab Y.H.A. (2022). Insulin secretory and antidiabetic actions of *Heritiera fomes* bark together with isolation of active phytomolecules. PLoS ONE.

[31] Gallagher A.M., Flatt P.R., Duffy G., Abdel-Wahab Y.H.A. (2003). The Effects of Traditional Antidiabetic Plants on in Vitro Glucose Diffusion. Nutr. Res..

[32] Ansari P., Hannon-Fletcher M.P., Flatt P.R., Abdel-Wahab Y.H.A. (2021). Effects of 22 Traditional Anti-Diabetic Medicinal Plants on DPP-IV Enzyme Activity and Glucose Homeostasis in High-Fat Fed Obese Diabetic Rats. Biosci. Rep..

[33] Hannan J.M.A., Ali L., Khaleque J., Akhter M., Flatt P.R., Abdel-Wahab Y.H.A. (2012). Antihyperglycaemic activity of *Asparagus racemosus* roots is partly mediated by inhibition of carbohydrate digestion and absorption, and enhancement of cellular insulin action. Brit. J. Nutr..

[34] Hannan J.M.A., Ansari P., Haque A., Sanju A., Huzaifa A., Rahman A., Ghosh A., Azam S. (2019). Nigella Sativa Stimulates Insulin Secretion from Isolated Rat Islets and Inhibits the Digestion and Absorption of (CH2O)N in the Gut. Biosci. Rep..

[35] Hannan J.M.A., Marenah L., Ali L., Rokeya B., Flatt P.R., Abdel-Wahab Y.H.A. (2007). Insulin secretory actions of extracts of *Asparagus racemosus* root in perfused pancreas, isolated islets and clonal pancreatic β-cells. J. Endocrinol..

[36] Azad S., Ansari P., Azam S., Hossain S., Shahid M.I.-B., Hasan M., Hannan J.M.A. (2017). Anti-Hyperglycaemic Activity of Moringa Oleifera Is Partly Mediated by Carbohydrase Inhibition and Glucose-Fibre Binding. Biosci. Rep..

[37] Hannan J.M.A., Ali L., Khaleque J., Akhter M., Flatt P.R., Abdel-Wahab Y.H.A. (2006). Aqueous extracts of husks of *Plantago ovata* reduce hyperglycaemia in type 1 and type 2 diabetes by inhibition of intestinal glucose absorption. Brit. J. Nutr..

[38] Amin M.N. (2019). In-Vivo Evaluation of Anti-Onciceptive, Anti-Inflammatory, Antipyretic, Hypoxia and Gastro-Intestinal Potentials of SwasKas Chintamani Ras. Discov. Med..

[39] Gupta R.K., Kesari A.N., Diwakar S., Tyagi A., Tandon V., Chandra R., Watal G. (2008). In vivo evaluation of anti-oxidant and anti-lipidimic potential of *Annona squamosa* aqueous extract in Type 2 diabetic models. J. Ethnopharmacol..

[40] Shirwaikar A., Rajendran K., Kumar C.D., Bodla R. (2004). Antidiabetic activity of aqueous leaf extract of *Annona squamosa* in streptozotocin–nicotinamide type 2 diabetic rats. J. Ethnopharmacol..

[41] Sharma A., Chand T., Khardiya M., Yadav K.C., Mangal R., Sharma A. (2013). Antidiabetic and antihyperlipidemic activity of *Annona squamosa* fruit peel in streptozotocin induced diabetic rats. Int. J. Toxicol. Pharmacol..

[42] Hannan J.M.A., Ali L., Rokeya B., Khaleque J., Akhter M., Flatt P.R., Abdel-Wahab Y.H.A. (2007). Soluble Dietary Fibre Fraction OfTrigonella Foenum-Graecum(Fenugreek) Seed Improves Glucose Homeostasis in Animal Models of Type 1 and Type 2 Diabetes by Delaying Carbohydrate Digestion and Absorption, and Enhancing Insulin Action. Brit. J. Nutr..

[43] Attiq A., Jalil J., Husain K. (2017). Annonaceae: Breaking the wall of inflammation. Front. Pharmacol..

[44] Billington C.K., Ojo O.O., Penn R.B., Ito S. (2013). CAMP Regulation of Airway Smooth Muscle Function. Pulm. Pharmacol. Ther..

[45] Chang L., Chiang S.-H., Saltiel A.R. (2004). Insulin signaling and the regulation of glucose transport. Mol. Med..

[46] Karlsson H.K.R., Zierath J.R. (2007). Insulin Signaling and Glucose Transport in Insulin Resistant Human Skeletal Muscle. Cell Biochem. Biophys..

[47] Chen H.-M., Lee L.-C., Hu K.-Y., Tsai W.-J., Huang C., Tsay H.-J., Liu H.-K. (2018). The application of post-translational modification oriented serum proteomics to assess experimental diabetes with complications. PLoS ONE.

[48] Flatt P.R., Abdel-Wahab Y.H.A., Boyd A.C., Barnett C.R., O’Harte F.P. (1997). Pancreatic B-cell dysfunction and glucose toxicity in non-insulin-dependent diabetes. Proc. Nutr. Soc..

[49] Vlassara H., Uribarri J. (2013). Advanced Glycation End Products (AGE) and Diabetes: Cause, Effect, or Both?. Curr. Diabetes Rep..

[50] Abdel-Wahab Y.H.A., Marenah L., Flatt P.R., Conlon J.M. (2007). Insulin releasing properties of the temporin family of antimicrobial peptides. Protein Pept. Lett..

[51] Das S., Das S., De B. (2012). In Vitro Inhibition of Key Enzymes Related to Diabetes by the Aqueous Extracts of Some Fruits of West Bengal, India. Curr. Res. Nutr. Food Sci..

[52] Gupta R.K., Kesari A.N., Watal G., Murthy P.S., Chandra R., Maithal K., Tandon V. (2005). Hypoglycemic and Antidiabetic Effect of Aqueous Extract of Leaves of *Annona squamosa* (L.) in Experimental Animal. Curr. Sci..

[53] Ding Y., Zhang Z., Dai X., Jiang Y., Bao L., Li Y., Li Y. (2013). Grape Seed Proanthocyanidins Ameliorate Pancreatic Beta-Cell Dysfunction and Death in Low-Dose Streptozotocin- and High-Carbohydrate/High-Fat Diet-Induced Diabetic Rats Partially by Regulating Endoplasmic Reticulum Stress. Nutr. Metab..

[54] Barnett A. (2006). DPP-4 inhibitors and their potential role in the management of type 2 diabetes. Int. J. Clin. Pract..

[55] Mizokami A., Yasutake Y., Gao J., Matsuda M., Takahashi I., Takeuchi H., Hirata M. (2013). Osteocalcin induces release of glucagon-like peptide-1 and thereby stimulates insulin secretion in mice. PLoS ONE.

[56] Zander M., Madsbad S., Madsen J.L., Holst J.J. (2002). Effect of 6-week course of glucagon-like peptide 1 on glycaemic control, insulin sensitivity, and β-cell function in type 2 diabetes: A parallel-group study. Lancet.

[57] Deacon C.F. (2019). Physiology and Pharmacology of DPP-4 in Glucose Homeostasis and the Treatment of Type 2 Diabetes. Front. Endocrinol..

[58] Zhang L., Zhang S.-T., Yin Y.-C., Xing S., Li W.-N., Fu X.-Q. (2018). Hypoglycemic effect and mechanism of isoquercitrin as an inhibitor of dipeptidyl peptidase-4 in type 2 diabetic mice. RSC Adv..

[59] Kashiwada M., Nakaishi S., Usuda A., Miyahara Y., Katsumoto K., Katsura K., Terakado I., Jindo M., Nakajima S., Ogawa S. (2021). Analysis of Anti-Obesity and Anti-Diabetic Effects of Acacia Bark-Derived Proanthocyanidins in Type 2 Diabetes Model KKAy Mice. J. Nat. Med..

[60] Gupta A., Jacobson G.A., Burgess J.R., Jelinek H.F., Nichols D.S., Narkowicz C.K., Al-Aubaidy H.A. (2018). Citrus Bioflavonoids Dipeptidyl Peptidase-4 Inhibition Compared with Gliptin Antidiabetic Medications. Biochem. Biophys. Res. Commun..

[61] Mathijs I., Da Cunha D.A., Himpe E., Ladriere L., Chellan N., Roux C.R., Joubert E., Muller C., Cnop M., Louw J. (2014). Phenylpropenoic acid glucoside augments pancreatic beta cell mass in high-fat diet-fed mice and protects beta cells from ER stress-induced apoptosis. Mol. Nutr. Food Res..

[62] Kiela P.R., Ghishan F.K. (2016). Physiology of Intestinal Absorption and Secretion. Best Pract. Res. Clin. Gastroenterol..

[63] Ahmed O.M., Moneim A.A., Yazid I.A., Mahmoud A.M. (2010). Antihyperglycemic, Antihyperlipidemic and Antioxidant Effects and the Probable Mechanisms of Action of Ruta Graveolens Infusion and Rutin in Nicotinamide-Streptozotocin-Induced Diabetic Rats. Diabetol. Croat..

[64] Müller M., Canfora E.E., Blaak E.E. (2018). Gastrointestinal Transit Time, Glucose Homeostasis and Metabolic Health: Modulation by Dietary Fibers. Nutrients.

[65] Zahid M., Mujahid M., Singh P.K., Farooqui S., Singh K., Parveen S., Arif M. (2018). *Annona squamosa* linn. (Custard apple): An aromatic medicinal plant fruit with immense nutraceutical and therapeutic potentials (Review). Int. J. Pharm. Sci. Res..

[66] Kumar M., Changan S., Tomar M., Prajapati U., Saurabh V., Hasan M., Sasi M., Maheshwari C., Singh S., Dhumal S. (2021). Custard Apple (*Annona squamosa* L.) Leaves: Nutritional Composition, Phytochemical Profile, and Health-Promoting Biological Activities. Biomolecules.

[67] Desai N., Barhate C., Biyani S., Kulkarni S., Nagarsenker M. (2011). Quantitative Analysis of Flavonoids in *Annona squamosa* Leaf Extracts and Its Pellet Formulation by Validated High-Performance Thin-Layer Chromatographic Technique. J. Planar Chromatogr. Mod. TLC.

[68] Liaw C.-C., Yang Y.-L., Chen M., Chang F.-R., Chen S.-L., Wu S.-H., Wu Y.-C. (2008). Mono-Tetrahydrofuran Annonaceous Acetogenins from *Annona Squamosa* as Cytotoxic Agents and Calcium Ion Chelators. J. Nat. Prod..

[69] Wei M., Chai W.-M., Yang Q., Wang R., Peng Y. (2017). Novel Insights into the Inhibitory Effect and Mechanism of Proanthocyanidins from *Pyracantha fortuneana* Fruit on α-Glucosidase. J. Food Sci..

[70] Li Y.Q., Zhou F.C., Gao F., Bian J.S., Shan F. (2009). Comparative Evaluation of Quercetin, Isoquercetin and Rutin as Inhibitors of Alpha-Glucosidase. J. Agric. Food Chem..

[71] Esmaeili M., Zohari F., Sadeghi H. (2009). Antioxidant and Protective Effects of Major Flavonoids from *Teulimacrium polium* on *β*-Cell Destruction in a Model of Streptozotocin-Induced Diabetes. Planta Med..

[72] Yokozawa T., Cho E.J., Park C.H., Kim J.H. (2011). Protective Effect of Proanthocyanidin against Diabetic Oxidative Stress. Evid.-Based Complement. Altern. Med..

[73] Neske A., Ruiz Hidalgo J., Cabedo N., Cortes D. (2020). Acetogenins from Annonaceae Family. Their Potential Biological Applications. Phytochemistry.

[74] Lima L.A.R.S., Pimenta L.P.S., Boaventura M.A.D. (2010). Acetogenins from Annona Cornifolia and Their Antioxidant Ca pacity. Food Chem..

